# Visual attention and recall of flavored cigarillo package elements among young adults: A randomized control trial

**DOI:** 10.1371/journal.pgph.0003840

**Published:** 2024-11-27

**Authors:** Stephanie Pike Moore, Alysha C. Ennis, Sho Kirihara, Elvia C. Gomez, Maya Reyes-Klein, Hannah Sharp, Joseph M. Macisco, Erika S. Trapl, Amanda J. Quisenberry, Elizabeth G. Klein

**Affiliations:** 1 Department of Population and Quantitative Health Sciences, School of Medicine, Case Western Reserve University, Cleveland, Ohio, United States of America; 2 College of Public Health, Ohio State University, Columbus, Ohio, United States of America; 3 College of Science, University of Nevada, Reno, Nevada, United States of America; 4 Allegheny College, Meadville, Pennsylvania, United States of America; 5 Department of Health Behavior, Roswell Park Comprehensive Cancer Center, Buffalo, New York, United States of America; University of Georgia College of Public Health, UNITED STATES OF AMERICA

## Abstract

**Introduction:**

Flavoring in cigarillos contributes to greater product initiation and abuse liability particularly among young adults. Few studies have examined how packaging elements, including flavor, may draw visual attention from potential consumers as well as impact product recall. This study aimed to test the difference in visual attention to flavor names and other packaging elements on cigarillo products as well as recall of these packaging elements.

**Materials and methods:**

Young adults aged 16–28 years were recruited to participate in a randomized control trial (Clinictrials.org ID: NCT04358705) in June through December 2022 (N = 89). Participants were randomized to two conditions viewing 12 flavored or unflavored cigarillo package images. Eye tracking software captured participants viewing (dwell) time on package features: flavor, brand, health warning, price, and other package imagery. Participants were asked about their recall of the images viewed. The proportion of dwell time on each package elements were used to examine differences between experimental conditions.

**Results:**

Across conditions, participants spent the greatest proportion of dwell time looking at the health warning. Participants in the flavored condition spent a significantly greater proportion of dwell time looking at flavor name (mean: 10.66%; SD: 3.56) compared to those in the unflavored condition (mean:7.03%; SD 2.81). Following the experiment, 45.8% of those in the flavored condition recalled having seen flavors or flavor names, which was greater than recall of all other elements and was significantly greater than flavor recall in the unflavored condition (12.2%).

**Conclusions:**

Flavored cigarillos attract greater visual attention and were the most memorable element of cigarillo packaging among young adults sampled. Changes to cigarillo packaging, including limiting flavor descriptors, imagery, and/or color on packaging and advertisements may be an effective way to reduce young adults’ attention to the products and thus their appeal.

## Introduction

Following the U.S. Family Smoking Prevention and Tobacco Control Act in 2009 [1], which banned the sale of flavored cigarette products, sales of other flavored tobacco products, such as flavored cigar products increased by nearly 50% by 2015 [2]. Since then, unit sales of cigarillos (i.e., a short, narrow cigar that contains ~3 grams of tobacco which are commercially mass produced and include brands such as Swisher Sweets and Black & Mild) [3], which account for 94.2% of cigar market share, continue to increase while all other cigar product sales (e.g.,. large and little cigars) have declined [4]. Furthermore, over half (53.3%) of cigarillo sales in 2020, up from 45.0% in 2009, were for products available in or marketed as being explicitly or implicitly flavored [5]. The overwhelming availability and marketing of flavored cigarillos have been a major contributing factor for younger populations to initiate and sustain product use [6]. Estimates suggest that 36.8% of young have ever used cigarillos by age 6 and 63.4% of them report that their first product was flavored [7]. Wave 2 of the Population Assessment of Tobacco and Health Study (PATH) show that among young adults who have used cigarillos in the past 12-months, 43.3% initiated cigarillo use with a flavored product [6]. Cigarillo flavors not only drive initiation, but young adults who demonstrate a preference for flavors exhibit greater symptoms of nicotine dependence [8]. Lastly, there are notable disparities reflecting the historic and pervasive marketing of cigarillos disproportionately to minoritized populations, particularly those who identify as Black or African American or as sexual and gender minorities, who experience greater prevalence of use of these products [9–11]. These factors, taken together, highlight the impact cigarillo flavors may have on health equity.

Product packaging conveys an abundance of information regarding the product itself. Across top-selling cigar products, products are consistently characterized by smaller package sizes (e.g., fewer individual product units), unique materials (e.g., re-sealable foil pouches to maintain freshness), flavor names, colors, imagery, and price promotions [12]. Each of these elements are intended to appeal to and impact potential consumer perceptions. For example, Delnevo et al. found that cigar package color and brand influenced how young adult consumers thought the product would taste or smell [13]. Price promotions, while decreasing the perceived quality of a product, increased intentions to purchase [14,15]. Packaging elements such as flavor or quality descriptors (e.g., “natural”) have been shown to increase favorable perceptions of products [14,16,17]. Across all package design elements, flavor was the most favorable and appealing element reported among adolescent and young adult consumers [16]. Jeong et al. found that even in cigarillo packaging with the same flavor, there are differences in appeal based on different descriptors, i.e. “boozy watermelon” vs “island madness” to describe rum-watermelon flavored cigarillos [15]. Sterling et al. describe that flavored cigar products specifically appeal to young adults due to a combination of packaging elements including color, imagery, and other smell/taste descriptors [18].

While the perception of various packaging elements related to cigarillos has been examined through numerous qualitative studies or studies involving self-report, few objective methods have been used to capture the important biobehavioral implications of package features. Measuring precise visual attention to various packaging elements to evaluate which details may consciously or unconsciously draw in consumers or potential consumers represents an important research gap. Eye tracking is a biobehavioral method to understand visual attention to minor details in packaging differences, allowing researchers to develop objective and direct measures of attention that may not be obtained through self-reported measures [19–21]. To date, several eye tracking studies conducted have focused specifically on packaging attributes across a number of tobacco products, predominantly cigarettes and electronic nicotine delivery systems (ENDS) [22–26]. Yet, to our knowledge, only one study has been conducted that has used this biobehavioral method to capture visual attention to cigarillo packaging elements which predominantly focused on visual attention to warning labels [27]. Given the preponderance of flavors and other packaging elements unique to cigarillos, there is a significant need to explore these features objectively.

The purpose of this research was to test the difference in visual attention to flavor names and other packaging elements for flavored and unflavored cigarillo products. Our primary hypothesis was that individuals who are shown cigarillo product packages marketed with explicit or implicit flavors would have a greater amount of visual attention to a package’s flavor name compared to those shown cigarillo product packages that are not marketed with an explicit or implicit flavor. We also hypothesized that participants would have greater recall for flavored product packaging features following the experiment.

## Materials and methods

### Study sample

**Fig 1:**
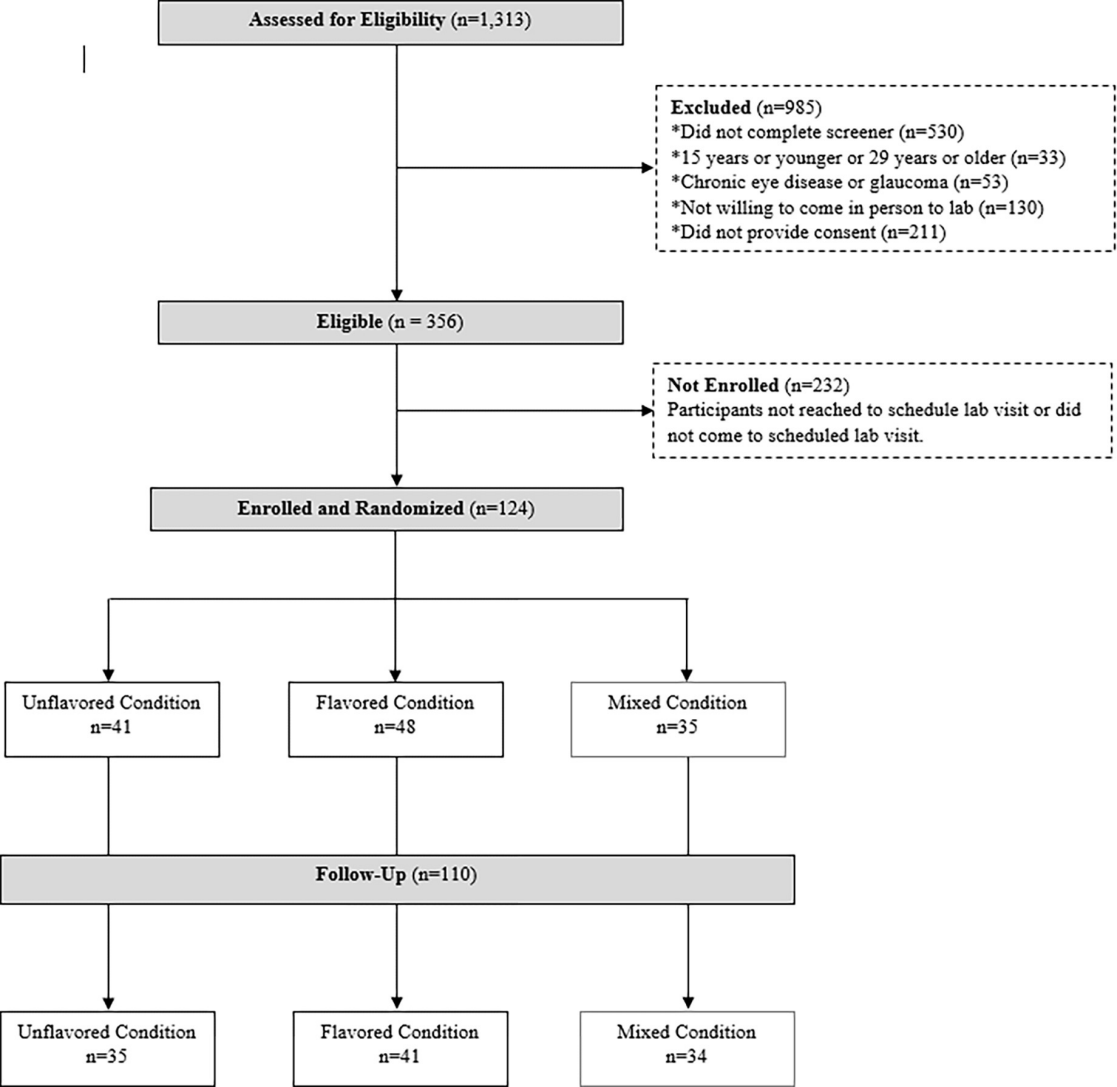
CONSORT diagram. Participants in the mixed condition were excluded from this analysis to better understand the impact of the implications of visual attention to flavored packaging and participant recall.

A convenience sample of young adults was recruited between May 9, 2022 and February 1, 2023 through social media and online outlets popular with the target age group (e.g., Reddit, Facebook, Instagram, ResearchMatch.org, GroupMe, etc.). Eligibility criteria included 1) being between the ages of 16 and 28 years of age; 2) self-report of having no chronic eye diseases known to interfere with calibration on eye tracking equipment such as glaucoma, regression lenses, or other similar issues; 3) being willing to participate in person for a session held in Columbus, Ohio; and 4) willing to complete online informed consent and participate in-person on the study protocol. Efforts were made to balance those who reported use of any cigarillos in the past 30 days (users) with those who reported no use in the past 30 days (non-users). A total of 1,313 unique individuals were screened, 356 met eligibility criteria, and 124 were enrolled and computer-randomized to one of three unblinded experimental conditions using eye tracking to examine flavored, unflavored, or a mixture of both flavored and unflavored cigarillo packages (Fig 1). Of these, 110 (88.7%) completed a follow-up survey one week after completing the eye tracking procedures. CONSORT checklist is located in S1 Checklist.

### Ethics statement

All participants in this study provided written informed consent as part of the web-based screening process once eligibility had been determined. The study protocol was reviewed and approved by The Ohio State University (OSU) Institutional Review Board (IRB) and was registered (Clinictrials.org ID: NCT04358705, “C-FLASH (Cigarillo Flavor and Abuse Liability, Attention, and Substitution in youtH)”) and described in S1 Protocol.

### Experimental procedures

Participants attended a single in-person eye tracking session in a private office space located in Columbus, OH at the Center for Tobacco Research. Participants were seated in a chair within a typical viewing distance from a monitor equipped with an infrared camera to capture precise eye movements using the Smart Eye Aurora [28]. A standard calibration procedure was completed to ensure data quality. During the experiment, participants had their gaze monitored continuously while a randomized set of stimuli (12 cigarillo product packages) were viewed for 6 seconds per image. This fixed interval was based on previous eye tracking studies that reported a mean viewing time of 5–10 seconds [29,30]. Following each package, an on-screen image re-centered the participant’s gaze for standardization prior to viewing the next image. Participants were shown images in a random sequence.

#### Experimental conditions

Eligible participants were randomly assigned to one of three image conditions: 1] 12 packages of cigarillo products marketed as having an explicit or implicit flavor (n = 41) herein referred to as the flavored condition, 2] 12 packages of cigarillo products not marketed as having an explicit or implicit flavor (n = 48) herein referred to as the unflavored condition, or 3] 6 packages of cigarillo products from the flavored condition and 6 packages of cigarillo products from the unflavored condition (n = 35) herein referred to as the mixed condition. Participants in the mixed condition were excluded from this analysis to better understand the impact of the implications of visual attention to flavored packaging and participant recall.

#### Experimental stimuli

Images of foil 2-packs of real-world cigarillos were identified from 12 current cigarillo brands. Product images for the flavored condition were selected based on the presence of marketing indicating explicit or characterizing flavors (e.g., blueberry, grape, honey, mango, peach, pineapple, wine) or marketing indicating implicit concept flavors (e.g., Kash, CaliGreen, Green Sweets which are all implicative of cannabis [31], Sweets, or Jazz). Product images for the unflavored condition were selected based on the absence of marketing indicating an explicit or implicit flavor based on descriptors, imagery, and package colors. Efforts were made to ensure that brands from the flavored condition were the same as or similar to the unflavored condition. However, given the preponderance of flavored cigarillos on the market [5], several brands in the flavored condition did not have a comparable product without explicit or implicit flavor characteristics.

A graphic designer modified the product packages for visual consistency so that all products were shown with identical pricing information (e.g., 2 for 99 cents) and a text-only health warning message was fixed on the bottom portion of the package for all products. Health warning messages were in accordance with the U.S. Food and Drug Administration’s (FDA) required warning statements [32].

#### Area of interest

**Fig 2:**
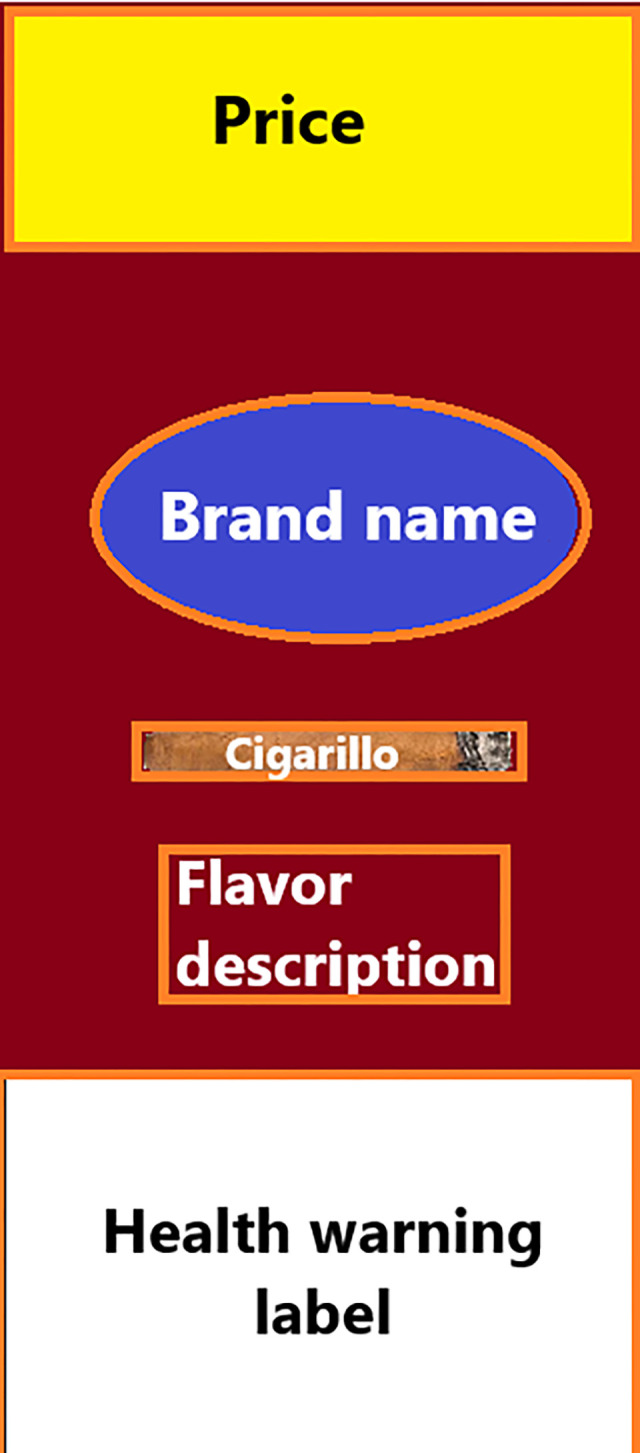
Unbranded example of each Area of Interest (AOI) where dwell time was captured for each image in both experimental conditions (outlined in orange). Attribution statement: Illustration created by Elizabeth Klein.

Dwell time, sometimes referred to as total fixation or fixation length, was calculated by aggregating the duration of fixations upon a defined area within a stimulus. The dwell time metrics, measured in milliseconds, were quantified by eye tracking equipment for specified areas of interest (AOI). Each AOI was selected based on the primary package descriptors including the product’s flavor name, brand, price promotion, health warning or package imagery including the image of a cigarillo (Fig 2). For unflavored product images, the product’s name (e.g., Classic, Black Label) was used to represent the flavor name. The proportional value for visual attention was calculated as the amount of dwell time spent within each defined AOI divided by the duration of time spent examining that image. The proportion of each AOI was measured by the eye tracking equipment as size (in percentage) divided by the entire cigarillo package for standardization across all cigarillo package stimuli. Differences in the respective size of each AOI with respect to each package image were evaluated to determine if there were differences by flavored and unflavored packages. No differences in the proportion of space occupied by select AOIs. On average, flavor names represented 4.3% of the total package when present, brand names were larger at 9.8% of space, price promotions took up 9.3% of the space, health warnings predominantly took up 28.8%, and cigarillo imagery 7.8% when present.

#### Image recall

Immediately following the experimental procedures and one week later, participants were asked to complete a web-based survey that asked open-ended questions prompting them to describe anything that they remembered seeing in the images from the experiment using as much detail as possible. Responses were classified based on aspects of the cigarillo packaging that the participant mentioned including flavors or flavor names (e.g., “Most were of cigarellos [sic], mostly flavored as a fruit flavor, but with some having names such as ’California Green" and other non-fruit flavors…” or “Lots of different brands had similar flavor choices, such as a "classic", "silver", or "Tobacco"…”), brands or brand names (e.g., “There were many packages for cigars each from a different brand…” or “The [Brand] and [Brand] are the only brands I recall..”, price promotions or product costs (e.g., “All of the packaging had a 2 for 99 cent deal…” or “There were also usually deals/sales on the top that were advertised for buying cigarillos in multiples for cheaper.”, health warnings (e.g., “…the thing that stood out to me the most was the warning label at the bottom”), imagery (e.g., “one of the packages had an owl on it”), other descriptors (“each product also indicated the flavor, and most said they had smooth and slow burn, as an appeal to the smoker…”), and package colors excluding colors used to describe price or health warnings (e.g., “I noticed the packages with unique colors, like the burgundy one…” or “cigarillo in dark packaging”).

### Statistical analysis

Bivariate analyses were used to examine demographic characteristics across experimental conditions using chi-square and Fisher’s Exact Tests. Bivariate analyses using t-tests were used to examine differences in the proportion of package allocated to each AOI, proportional dwell time for each AOI across both conditions as well as across individuals who reported use of cigarillos in the past 30 days compared to those who had not used any in the past 30 days. Independent paired t-tests were used to evaluate within-subject differences of those in the flavored condition with respect to proportional dwell time for cigarillos with explicit or characterizing flavors and those with implicit or concept flavors.

## Results

On average, participants of this study were 20.9 years of age (range: 17–28 years), 51.7% identified as female, 78.7% identified as heterosexual 14.6% identified as bisexual or pansexual, 78.7% were White or Caucasian and 7.9% were Black or African American, 4.5% were Hispanic, 70.8% had some college or an associate degree, and 29.2% had used cigarillos in the past 30 days (Table 1). There were no differences in age, gender, racial or ethnic identity, or use of cigarillos in the past 30-days across both study conditions.

**Table 1:** Young adult sample demographic characteristics by experimental condition.

	Analytic Sample		Unflavored Condition^a^		Flavored Condition^a^		p-value
	N = 89		n = 41		n = 48		
	N/mean	%/SD	n/mean	%/SD	n/mean	%/SD	
**Age**	20.9	2.4	21	2.3	20.8	2.5	0.6173
**Gender**							0.9346
Female	46	51.7	22	53.7	24	50.0	
Male	39	43.8	17	41.5	22	45.8	
Genderqueer/Nonbinary	4	4.5	2	4.9	2	4.2	
**Sexual Orientation**							0.3856
Heterosexual	70	78.7	31	75.6	39	81.3	
Gay, Lesbian, Queer	2	2.2	0	0.0	2	4.2	
Bisexual/Pansexual	13	14.6	7	17.1	6	12.5	
Unsure	4	4.5	3	7.3	1	2.1	
**Race**							0.5243
White or Caucasian	70	78.7	30	73.2	40	83.3	
Black or African American	7	7.9	5	12.2	2	4.2	
Asian	8	9.0	4	9.8	4	8.3	
Multiracial	4	4.5	2	4.9	2	4.2	
**Ethnicity**							1.0000
Hispanic	4	4.5	2	4.9	2	4.2	
**Educational Attainment**							0.7153
High School or Less	6	6.7	2	4.9	4	8.3	
Some College or Associate’s Degree	63	70.8	31	75.6	32	66.7	
Bachelors Degree or More	20	22.5	8	19.5	12	25.0	
**Past 30-Day Cigarillo Use**	26	29.2	12	29.3	14	29.2	0.9320
**Completed Follow-Up**	76	85.4	35	85.4	41	85.4	1.0000

^a^Those in the flavored condition were shown 12 packages of cigarillos that are marketed with an explicit or implicit flavor; those in the unflavored condition were shown 12 packages of cigarillos that are not marketed with an explicit or implicit flavor.

On average, the first AOI viewed across all packages, most frequently (19.2%) was of images depicting the shape of a cigarillo(s) when such an image was present. This was followed by brand name (16.9%), health warning (13.4%), and flavor name (13.0%) (Table 2). No differences were observed between those in the flavored or unflavored condition in which AOI was viewed first.

**Table 2:** Eye-tracking metrics among young adults viewing flavored and unflavored cigarillo product packages.

	Flavored Condition^a^			Unflavored Condition^a^			p-value^b^
	n = 48			n = 41			
	Mean	SD	95% CI	Mean	SD	95% CI	
**First Fixation** ^c^							
Flavor Name^d^	12.5	6.5	(9.8, 15.2)	12.9	5.8	(10.6, 15.3)	0.8035
Cigarillo Brand	14.1	8.6	(11.2, 17.0)	18.8	14.4	(13.5, 24.1)	0.1198
Price Promotion	12.3	8.4	(8.9, 15.8)	12.3	8.9	(7.7, 16.8)	0.9770
Health Warning	15.5	8.6	(10.5, 20.4)	12.2	6.2	(8.8, 15.7)	0.2487
Cigarillo Image^e^	18.4	8.2	(15.7, 21.1)	20.7	10.8	(15.6, 25.8)	0.3708
**Proportion of Dwell Time** ^f^							
Flavor Name^d^	10.7	3.6	(9.6, 11.7)	7.0	2.8	(6.1, 7.9)	<0.001
Cigarillo Brand	13.2	4.4	(11.9, 14.4)	16.7	7.0	(14.5, 18.9)	0.0072
Price Promotion	10.4	5.7	(8.7, 12.0)	8.6	5.5	(6.9, 10.4)	0.1556
Health Warning	22.2	14.1	(18.1, 26.3)	24.2	13.8	(19.9, 28.6)	0.4916
Cigarillo Image^e^	10.6	4.3	(9.3, 11.8)	8.8	5.4	(7.1, 10.5)	0.0843

^a^Those in the flavored condition were shown 12 packages of cigarillos that are marketed with an explicit or implicit flavor; those in the unflavored condition were shown 12 packages of cigarillos that are not marketed with an explicit or implicit flavor.

^b^Based on bivariate analyses using t-tests. For flavor type, independent paired sample t-tests were conducted to compare within-subject differences in proportional dwell time to flavor names across characterizing and concept flavors.

^c^The mean represents the average number of times a study participant’s first fixation was within the specified area of interest across all images.

^d^Flavor name was not provided on one unflavored cigarillo package.

^e^Cigarillo images were available on 10 of the flavored cigarillo packages and 7 of the unflavored cigarillo packages.

^f^The mean represents the average proportional dwell time measured as the sum of total dwell time spent within the specified area of interest divided by the total amount of time the image was displayed.

Across both conditions, the greatest proportional dwell time was spent within the health warning AOI with 22.2% (SD: 14.1) and 24.2% (SD:13.8) of dwell times in the flavored and unflavored conditions, respectively (Table 2). Those in the flavored condition spent a greater proportional dwell time viewing the flavor name compared to those in the unflavored condition (10.7% (SD:3.7), and 7.0% (SD:2.8), respectively). Those in the unflavored condition spent a greater proportional dwell time viewing price than those in the flavored condition (16.7% (SD: 7.0) compared to 13.2% (SD:4.4)). Within the flavored condition, participants spent a significantly greater proportion of dwell time on concept flavor names compared to characterizing flavor names, on average (12.1% (SD: 4.5) and 9.6% (SD: 3.6), respectively, p<0.001) based on an independent paired t-test.

In examining individual images, flavored products such as CaliGreen and Blueberry had the greatest proportional dwell time (22.5%, and 21.2%, respectively) (S1 Table). These images, notably, have the flavor name listed twice on the packaging. However, with respect to unflavored products, the greatest proportional dwell time was spent examining Natural Buzz (16.3%), De Luxe (15.7%) and Black Label (14.4%).

There were differences in the proportion of dwell time observed within each condition by cigarillo use history (S2 Table). Among those in the flavored condition, those who used a cigarillo in the past 30 days had greater dwell time examining flavor name (12.4% (95% CI: 9.9, 14.9) compared to those who had not used cigarillos (10.0% (95% CI: 8.9, 11.0)). Among both conditions non-users had a greater dwell time for health warnings compared to those who had used cigarillos.

Immediately following the experiment, 45.8% of those in the flavored condition recalled view flavors or a specific flavor compared to those in the unflavored condition 12.2% (p<0.001) (Table 3). No other differences were observed across condition and recall of packaging elements immediately following the experiment The same differences in recall were retained at one-week follow-up.

**Table 3:** Young adult recall of image elements after eye-tracking study^a^.

	Flavored Condition^b^		Unflavored Condition^b^		p-value^c^
	n = 48		n = 41		
	%	95% CI	%	95% CI	
**Recall Element**					
Flavor or Flavor Name	45.8	(31.2, 60.5)	12.2	(1.7, 22.7)	0.0010
Brand or Brand Name	33.3	(19.5, 47.2)	26.8	(12.7, 41.0)	0.5059
Price or Product Cost	41.7	(27.2, 56.1)	39.0	(23.4, 54.6)	0.8001
Health Warning	52.1	(37.4, 66.7)	46.3	(30.4, 62.3)	0.5892
Imagery	25.0	(12.3, 37.7)	19.5	(6.8, 32.2)	0.6154
Descriptor	18.8	(7.3, 30.2)	9.8	(0.3, 19.2)	0.3672
Color^d^	29.2	(15.8, 42.5)	19.5	(6.8, 32.2)	0.3326

^a^Recall was based on an open-ended question about what participants remembered seeing during the experimental phase immediately following the experiment.

^b^Those in the flavored condition were shown 12 packages of cigarillos that are marketed with an explicit or implicit flavor; those in the unflavored condition were shown 12 packages of cigarillos that are not marketed with an explicit or implicit flavor.

^c^Based on bivariate analyses using chi-square and Fisher’s exact tests.

^d^ Exlcudes recall of the colors related to price promotions or health warning labels.

## Discussion

In this eye tracking study using real-world cigarillo products, we found that young adult participants spent a greater proportion of dwell time on the cigarillo flavor name in the flavored condition compared to those in the unflavored condition (10.8% vs 7.0%, p<0.001). This suggests that explicit flavor names (e.g. honey, grape, peach) or implicit flavor names (e.g., CaliGreen), capture the visual attention of young adults to a greater degree than those without explicit or implicit flavor names (e.g. original, silver, brown). This finding is consistent with our hypothesis that products marketed with an explicit or implicit flavor would attract a greater amount of visual attention compared to products that do not have an explicit or implicit flavor Differences across conditions were also seen with respect to brand, with more dwell time seen on brand in the unflavored condition (16.7% vs 13.2%, p = 0.0072). No other differences were observed across conditions concerning other packaging elements such as price, warning, or cigarillo imagery.

Our findings are in line with Londerée et al. who similarly found greater visual attention to flavored electronic nicotine delivery systems (ENDS) compared to unflavored ENDS when viewing real-world point-of-sales advertisements [33]. Furthermore, our findings are also consistent with other studies of this age group showing a preference for fruit and/or sweet flavored tobacco products [14,34]. Notably, our study did not show any differences in visual attention to health warnings by flavored condition, which has been suggested in Garrison et al. exploratory eye tracking study of e-cigarette products [35].

Those in the unflavored condition spent more time looking at the brand name than those in the flavored condition. Brand preference remains an important element for individuals who use cigarillo products [13,25,36]. This study highlights that branding may also impact individuals who are non-users of cigarillos. While the present study used 12 cigarillo brands, including those with lower market share [2,5,37], the brand names were largely comparable across conditions. This difference may indicate the influence of brand recognition or brand preference and highlights that, in the absence of explicit or implicit flavor names, branding may become a focal point. Notably, however, visual attention to health warnings still represents the greatest proportional dwell time although health warnings represented 28.8% of packaging space on average (range 22.6% to 38.0%). Maynard et al. found that in the absence of packaging elements on cigarette packs (e.g., plain packaging) visual attention to health warnings may drive attention away from brand names [25].

Recall is an important metric, especially to assess receptivity among susceptible nonusers of tobacco products [38,39]. Recall has been predominantly studied in the context of health warnings highlighting that color, size, or pictorial representations rather than text-only messages may improve health warning recall and reduce recall of other packaging elements such as brand [27,40,41]. While there were no differences observed in recall of health warning labels across experimental conditions, health warnings were recalled by about half of participants in each condition both immediately following and one week later. The only observable difference across conditions specific to recall was related to product flavors or flavor names which represented the element with the greatest recall among those in the flavored condition aside from health warnings. No other differences were observed in recall with any other element including price promotions, branding, health warnings, package colors, imagery, or descriptors. Thus, exposure to flavored cigarillo packaging may have important implications potentially influencing young adult consumer perceptions and behavior.

This research highlights that flavors attract a greater amount of visual attention and are one of the most memorable packaging traits. These results, taken with the abundance of research indicating that flavors appeal to younger populations [16,18,42,43] and may be a unique addictive attribute that sustains use [6,8], highlight an important opportunity for tobacco control from both the lens of prevention and the lens of cessation. Restricting the use of color, flavor names, and flavor descriptions, or requiring the use of plain packaging, may provide a salient solution to reduce the visual attention and appeal of these flavored products. In the United Kingdom and European Union, cigarillos are notably exempt from plain packaging rules [44]. In the U.S., the proposed 2022 FDA restrictions on flavors in cigar products includes limiting flavor representation on a label or in marketing materials but these restrictions appear to only apply to those products with explicit characterizing flavors [45]. Excluding products with implicit, or concept flavors, represents a potential policy loophole [46]. Evidence suggests that the tobacco industry in the U.S. has already begun to shift toward implicit flavors making use of this loophole [44]. While differences were detected in visual attention to characterizing and concept flavors, this study was not powered to examine these differences thus highlighting an avenue for future research to examine how attention to these flavored products may differ.

Differences were observed within each condition based on past 30-day use of cigarillos wherein individuals who had not used cigarillos had significantly greater visual attention to health warnings compared to those who had used these products which reinforces the findings presented by Nonnemaker et al. Among those in the flavored condition, individuals who had used cigarillos had greater visual attention to flavor name and cigarillo imagery compared to those who had not used cigarillos [27]. However, due to limitations of the convenience sample, only 29% of our sample included consumers of cigarillos, which limits our interpretation of these findings. The small sample size of this group coupled with these findings represents an important area for future research to objectively assess these differences to determine if and how packaging elements may differentially impact these groups and highlight opportunities for both prevention and cessation.

This research presented in this study provides compelling evidence on visual attention to cigarillo packaging elements but several notable limitations that should be considered. The lab environment is not necessarily reflective of a real-world scenario where a young adult may view a variety of products at a convenience store and would be less likely to spend 8–10 seconds to review each individual product. Future eye tracking research could measure which AOIs young adults’ attention are drawn to when viewing an array of products together behind a counter. Behavioral economics research, such as an experimental tobacco marketplace, could similarly be used to simulate a more real-world purchasing scenario. Lastly, in the present study, some of the chosen cigarillo flavors could be considered as concept flavors, while the unflavored products were not; increased examination to the implicit message associated with flavor names and their meaning will be important to examine as the industry responds to regulatory changes around flavors [47].

## Conclusions

It is known that the brand and flavor names included on cigarillo packaging contribute to young people’s perceptions of and intentions to purchase cigarillos [13,16]. Our findings support that flavor names attract attention and retain recall over a short time period among young adults who do and do not use cigarillos. Changes to cigarillo packaging, which could include banning flavors or flavor descriptors, may be an effective way to reduce young adults’ attention to the products and thus their appeal. This adds to the growing body of research on cigarillos demonstrating the potential benefits of banning cigarillo flavors to reduce sales to youth and young adults [47].

## Supporting information

S1 Checklist CONSORT checklist. (DOC)

S1 Protocol Study protocol. (DOCX)

S1 Table Proportional dwell time for each experimental stimuli. (DOCX)

S2 Table Proportion of dwell time viewing flavored and unflavored cigarillo product packages by cigarillo use. (DOCX)
