# Supplementary material for: Visual attention and recall of flavored cigarillo package elements among young adults: A randomized control trial
Source: PLOS Glob Public Health. 2024 Nov 27;4(11):e0003840. doi: 10.1371/journal.pgph.0003840 (PMC11602028; doi:10.1371/journal.pgph.0003840)
Supplement: S1 Protocol — (DOCX) [file pgph.0003840.s002.docx]

**C.2. Approach for Aim 2**

**Aim 2. To examine the impact of flavored cigarillo products on visual attention and risk perceptions among youth and young adult cigarillo users and nonusers.** A between-subjects experiment will expose a convenience sample of cigarillo users and non-users (ages 16-28) to view a set of stimuli depicting cigarillo packaging. Participants will be randomly assigned to one of three conditions: 1) flavored only, 2) unflavored only, or 3) half flavored and half unflavored product packages for cigarillos. Participants will view each product package for 10 seconds while eye tracking equipment captures visual attention on the features displayed on-screen. Analyses will compare participants viewing the flavored cigarillo product (versus unflavored) on their visual attention to the cigarillo product, risk perceptions, and rating of product appeal. One week after the experiment, a follow-up survey will capture participant’s recall of cigarillo brand, presence of any cigarillo flavor (yes/no), recall of product colors and tobacco use behaviors and intentions.

**C.2.1. Participants.** We will recruit a convenience sample (n=150) based on the following eligibility criteria: 1) 16 years to 28 years of age, 2) not having chronic eye diseases known to interfere with eye tracking equipment (e.g., glaucoma, regression lenses, etc.), 3) willing to participate in person for a session held in Columbus, Ohio, and 4) being willing to provide informed consent and participate in the study protocol. An initial screening contact will determine eligibility and fulfill two stratification variables to balance the study sample: past month cigarillo use (yes/no) and age (16-21/22-28 years). Participants will be recruited through the social media and traditional advertisement channels popular with the target age group (e.g., Reddit, Facebook, Instagram, Reddit, ResearchMatch, university advertisements, etc.). Cigarillo users who dual use tobacco and/or cannabis will still be eligible to participate, but non-users will be ineligible if they report any tobacco use in the past month. Participants will be offered a $50 gift card for participation; those unable to be calibrated for the eye tracking equipment will be given a $5 gift card. Participants completing the follow-up survey online will be offered a $25 gift card.

**C.2.2. Study Procedures.** Participants will be deemed eligible at screening, followed by a single in-person eye tracking session in a private office space on the OSU campus. After informed consent procedures are conducted, participants will complete a pre-experimental survey using Qualtrics to measure cigarillo use history (users) or susceptibility (non-users) (see **Table 4** for specific measures). Next, a participant will be seated in a chair within a typical viewing distance from a monitor equipped with an infrared camera to capture precise eye movements (Smart Eye Aurora Tracker). A standard calibration procedure will be completed to assure data quality. During the experiment, participants will have their gaze monitored continuously while a randomized set of stimuli (product packages) are viewed for 10 seconds per image; this fixed interval is based on previous eye tracking studies reporting mean viewing time of 8-10 seconds.^63,85,86^ Following each package, an on-screen image will re-center the participant’s gaze for standardization prior to the next image. After conclusion of the experiment, participants will complete a post-experimental survey. One week following the completion of the experiment, all participants will be invited via email and/or text to complete an online follow-up online survey to measure recall of brand name, product flavors, product package colors, perceptions of cigarillo harm, and tobacco use behaviors and intentions. Data collection tools for the complete experiment are shown in Appendix B.

**C.2.3. Experimental Stimuli.** In addition to cigarillo packages, 2 e-cigarette liquids and 2 sports drinks images will be shown to each participant in a random sequence. Flavored foil 2-packs of cigarillos will be drawn from 12 current brands.^32^ A graphic designer will modify the product packages for visual consistency so that all products are shown with identical pricing information (e.g., 2 for 99 cents) and a text-only health warning message will be shown on the bottom portion of the package for all products. To evaluate color, half of the cigarillo products (n=6) will be modified to use black, white and grey as the primary package color (referred to here as plain packaging), with the other half of products using the originally designed colors for the product package.

Two factors will be examined within the set of images: flavor descriptors and colors. In the unflavored condition participants will view 16 imagers: 12 cigarillo products currently sold as unflavored (“original”, “classic”, etc) across 12 unique brands + 2 e-liquids in “tobacco” flavor +2 brands of unflavored sports drinks. In the flavored condition, participants will view 12 flavored cigarillos; half will be traditional flavors (grape, blueberry, etc.) and half will be concept flavors that do not represent foods or beverages (e.g., jazz, arctic rush, green, etc.). Participants will also view 2 e-liquids (1 traditional + 1 concept flavor) and 2 sports drinks (1 traditional flavor + 1 concept flavor). The blended condition will include half flavored (2 traditional + 1 concept flavor) and half unflavored products; selected brands for the blended condition will be based on dominant brands of in the region (e.g., Swisher Sweets, Black & Mild, etc.)

**C.2.4. Study measures.** The primary outcome measure will be mean value for *visual attention* (measured in milliseconds by eye tracking equipment) on the area of interest showing the cigarillo product (See **Table 4**). A secondary outcome of overall perception of risk (harm rating index^88^ and relative risk) of cigarillo products. Product appeal will be measured using a standard tobacco use measure modified for cigarillos.^88^ Future use intention will be measured following methods used by Pierce et al (response of “definitely no”/any other response).^89^ A summary of participant measures is shown in **Table 4**, including relevant measures from the PhenX Toolkit as requested in the FOA.

| **Table 4. Aim 2 Measures, Sources, and Response Categories** | | |
| --- | --- | --- |
| ***Measures*** | ***Source*** | ***Responses*** |
| *Visual attention Post viewing harm rating*  *Relative risk perception*  *Cigarillo appeal rating index*  *Unaided brand/product recall* | Eye tracking software  Sterling et al.  Vasiljevic et al.  Capella et al. | Dwell time [milliseconds]  Flavor text/imagery; product package; Brand/logo  How harmful is the product you just viewed? (not at all/extremely)  Compared to cigarettes, how harmful do you think flavored cigarillos are? (less/same/more/don’t know)  Compared to unflavored cigarillos, how harmful do you think flavored cigarillos are? (less/same/more)  Compared to e-cigarettes, how harmful do you think flavored cigarillos are? (less/same/more/don’t know)  Compared to unflavored/tobacco-flavored e-cigarettes, how harmful do you think flavored cigarillos are? (less/same/more)  How do you feel about using cigarillos? (unattractive-attractive, not cool-cool, boring-fun; rated 1-5)  How much did you like this product? (10-point scale: not at all/very much)  Please list any of the products you saw during the experiment; provide as much detail as you can (open-ended response) |
| *Cigarillo susceptibility*  *Product use intentions* | Pierce et al. | How likely are you to use cigarillos if your best friend offered you one? [non-users; 4-point scale of definitely yes-definitely no]  How likely are you to use cigarillos in the next year? [non-users]  How likely are you to use cigarillos soon? [non-users]  How likely are you to use cigarillos this week? [users; 4-point scale of definitely yes-definitely no] |
| *Flavor preferences*  *Tobacco use history*  *Smoking self-perception*  *Marijuana use* | PATH study | Rank flavors (i.e. fruit, sweet, candy, alcohol, mint, tobacco) from (1) best taste to worst taste; (2) least harmful to most harmful to health; (3) most natural to least natural  LCC, Cigarette, e-cigarette, smokeless tobacco use (ever)  Current tobacco product use (Most days, some days, not at all)  Do you consider yourself a smoker? (yes/no)  Have you ever used marijuana, hash, THC, or grass? (yes/no) |
| *Social factors*  *Smoking in the household*  *100% smoke-free home*  *Close friend tobacco use* | Current Population Survey, Tobacco Use Supplement | Current household tobacco use (parents, siblings)  Which statement best describes the rules about smoking in your home (none/some/anywhere)  How many of your 5 closest friends use tobacco? (0-5) |

**C.2.5. Statistical analysis.** Five study hypotheses will be tested:

H1: The proportion of viewing time on the product package will be greater for flavored compared to unflavored cigarillo packaging.

H2: Flavored cigarillos will receive higher appeal ratings and lower absolute and relative health risks compared to unflavored cigarillos.

H3: Flavored cigarillos will yield greater odds of future use intentions compared to unflavored cigarillos.

H4: (Exploratory) The proportion of viewing time on the cigarillo product packages with color will attract greater attention compared to the plain product packages.

H5: (Exploratory): The effect of flavor (H2-H3) will be modified by user status.

H6: (Exploratory): Concept flavors descriptors will attract greater visual attention than traditional flavor descriptors.

In our statistical analyses, we will report means and standard deviations for the primary outcome (dwell time visual attention on product packages) and other continuous outcomes. We will use a two-way ANOVA using a general linear model for repeated measures to test for the main effects of the presence of flavor in cigarillo products and the exploratory interaction between flavor and user status. We will use logistic regression to examine the product use intentions (any/none) at the one- week follow-up survey.

**C.2.6. Sample size justification.** The sample size was estimated based on previous eye tracking research looking at static content on screen that displayed tobacco products or advertising.^63,85,86^ We are assuming non- significant mean differences in visual attention between susceptible non-users and current cigarillo users and that mean difference is maintained when participants see both flavored and unflavored products. For the primary outcome of visual attention, we will estimate differences in mean dwell time between flavored and unflavored cigarillo packages. Within an individual viewing all flavored or all unflavored products, we assume a within-participant correlation of 0.5 for total viewing time of each tobacco product package, and we conservatively assume minimal correlation of dwell time between flavored and unflavored products within the same participant. Based on these factors, we have planned for 150 participants total (n=50 per study condition), and we will have 90% power to detect moderate effect size of 0.4 between flavored and unflavored conditions. These effects are consistent with the trends observed in previous research on eye tracking,^91^ and are large enough to support the examination of the secondary outcomes; the inclusion of non-users will allow exploration of the potential interaction between flavor and user status across all secondary and exploratory hypotheses.
