# Supplementary material for: Visual attention and recall of flavored cigarillo package elements among young adults: A randomized control trial
Source: PLOS Glob Public Health. 2024 Nov 27;4(11):e0003840. doi: 10.1371/journal.pgph.0003840 (PMC11602028; doi:10.1371/journal.pgph.0003840)
Supplement: S1 Table — (DOCX) [file pgph.0003840.s003.docx]

| **Supplemental Table 1. Proportional dwell time for each experimental stimuli** | | | | | | | | | | |
| --- | --- | --- | --- | --- | --- | --- | --- | --- | --- | --- |
|  | **Area of Interest** | | | | | | | | | |
|  | **Flavor Name** | | **Cigarillo Brand** | | **Price Promotion** | | **Health Warning** | | **Cigarillo Image** | |
|  | Mean | SD | Mean | SD | Mean | SD | Mean | SD | Mean | SD |
| **Flavored Condition^a^** |  |  |  |  |  |  |  |  |  |  |
| Grape | 3.77 | 1.61 | 16.79 | 10.25 | 11.83 | 10.01 | 21.96 | 16.06 | - | - |
| Blueberry | 21.18 | 15.20 | 12.24 | 9.22 | 11.17 | 8.27 | 22.14 | 17.15 | 9.22 | 7.09 |
| Wine | 6.99 | 4.42 | 14.74 | 9.35 | 8.31 | 6.92 | 23.85 | 16.45 | 10.00 | 8.37 |
| Peach | 7.23 | 5.42 | 10.65 | 5.63 | 11.65 | 6.47 | 26.57 | 17.18 | 7.94 | 5.10 |
| Mango | 10.07 | 4.67 | 23.24 | 11.81 | 12.85 | 15.05 | 32.89 | 18.54 | 47.07 | 17.27 |
| Honey | 10.99 | 9.22 | 11.65 | 8.74 | 34.67 | 19.01 | 21.32 | 18.12 | 12.34 | 16.28 |
| Pineapple | 17.17 | 12.69 | 11.16 | 6.61 | 13.34 | 8.04 | 24.32 | 19.31 | 8.28 | 6.48 |
| Kash | 15.61 | 8.26 | 10.47 | 6.49 | 13.07 | 8.15 | 30.57 | 17.52 | 6.55 | 4.47 |
| CaliGreen | 22.45 | 12.70 | 10.90 | 6.82 | 11.60 | 13.16 | 21.43 | 16.47 | 7.61 | 7.78 |
| Green Sweets | 7.35 | 5.53 | 14.63 | 7.54 | 13.03 | 10.95 | 26.42 | 19.25 | - | - |
| Sweets | 9.90 | 7.26 | 14.47 | 7.20 | 10.06 | 6.48 | 23.9 | 19.24 | 7.30 | 8.67 |
| Jazz | 11.48 | 5.21 | 22.64 | 12.71 | 0.70 | 11.53 | 27.95 | 21.98 | 17.08 | 10.41 |
| **Unflavored Condition^a^** |  |  |  |  |  |  |  |  |  |  |
| Classic (red package) | 3.60 | 2.06 | 14.12 | 7.55 | 19.98 | 12.24 | 28.37 | 22.21 | - | - |
| Black Label | 14.43 | 7.08 | 12.98 | 8.16 | 10.08 | 7.05 | 22.25 | 13.93 | 7.52 | 6.51 |
| Classic (brown package) | 7.17 | 4.47 | 24.82 | 14.64 | 8.76 | 4.78 | 36.68 | 19.54 | 18.73 | 10.92 |
| Straight Up | 6.46 | 7.44 | 19.44 | 11.51 | 10.64 | 7.51 | 26.1 | 20.81 | 7.50 | 4.80 |
| Silver (blue package) | 7.50 | 5.48 | 25.39 | 15.13 | 12.89 | 6.48 | 39.2 | 22.75 | - | - |
| Original (brown package) | 5.58 | 2.89 | 12.64 | 7.52 | 15.05 | 11.02 | 28.26 | 16.88 | 11.39 | 14.17 |
| Pure Silver | 9.24 | 8.53 | 8.16 | 5.79 | 9.53 | 6.77 | 27.22 | 18.39 | - | - |
| Silver (black package) | 8.71 | 7.26 | 23.08 | 12.51 | 11.52 | 7.81 | 31.29 | 18.61 | 14.57 | 14.18 |
| De Luxe | 15.66 | 10.73 | 24.78 | 16.40 | 10.23 | 5.81 | 21.67 | 16.63 | - | - |
| Brown | 6.96 | 4.94 | 20.01 | 10.52 | 15.35 | 13.40 | 21.89 | 14.02 | 15.15 | 11.07 |
| Original (silver package) | - | - | 17.33 | 10.59 | 12.09 | 6.36 | 23.67 | 17.97 | 4.84 | 2.86 |
| Natural Buzz | 16.25 | 7.83 | 18.90 | 10.83 | 15.29 | 11.30 | 30.63 | 18.47 | - | - |
| ^a^ Those in the flavored condition were shown 12 packages of cigarillos that are marketed with an explicit or implicit flavor; those in the unflavored condition were shown 12 packages of cigarillos that are not marketed with an explicit or implicit flavor. Cigarillo products were purchased by the authors and images were captured and modified by the authors.  Data notes: The mean represents the average proportional dwell time measured as the sum of total dwell time spent within the specified area of interest divided by the total amount of time the image was displayed. Cells denoted with - indicates that these package elements were not present in that product’s image. | | | | | | | | | | |
