# Supplementary material for: Visual attention and recall of flavored cigarillo package elements among young adults: A randomized control trial
Source: PLOS Glob Public Health. 2024 Nov 27;4(11):e0003840. doi: 10.1371/journal.pgph.0003840 (PMC11602028; doi:10.1371/journal.pgph.0003840)
Supplement: S2 Table — (DOCX) [file pgph.0003840.s004.docx]

| **S2 Table Proportion of dwell time viewing flavored and unflavored cigarillo product packages by cigarillo use** | | | | | | | | | | |
| --- | --- | --- | --- | --- | --- | --- | --- | --- | --- | --- |
|  | **Flavored Condition**^a^ | | | |  | **Unflavored Condition**^a^ | | | |  |
|  | **Used**  **Cigarillos**^b^ | | **Did not Use Cigarillos**^b^ | | p-value^c^ | **Used**  **Cigarillos**^b^ | | **Did not Use Cigarillos**^b^ | | p-value^c^ |
|  | n=14 | | n=34 | |  | n=12 | | n=29 | |  |
|  | **Mean** | **95% CI** | **Mean** | **95% CI** |  | **Mean** | **95% CI** | **Mean** | **95% CI** |  |
| **Proportional Dwell Time**^d^ |  |  |  |  |  |  |  |  |  |  |
| Flavor Name | 12.4 | (9.9, 14.9) | 10.0 | (8.9, 11.0) | 0.0320 | 7.0 | (4.6, 9.4) | 7.0 | (6.1, 7.9) | 0.9935 |
| Cigarillo Brand | 14.9 | (12.0, 17.9) | 12.5 | (11.1, 13.8) | 0.0724 | 17.9 | (11.5, 24.2) | 16.2 | (14.1, 18.2) | 0.5877 |
| Price Promotion | 9.9 | (7.4, 12.4) | 10.5 | (8.4, 12.7) | 0.7106 | 7.7 | (4.7, 10.6) | 9.0 | (6.8, 11.2) | 0.4647 |
| Health Warning | 15.2 | (10.1, 20.4) | 25.0 | (19.8, 30.2) | 0.0277 | 17.8 | (9.1, 26.2) | 26.9 | (21.9, 32.0) | 0.0490 |
| Cigarillo Image | 13.2 | (10.4, 16.0) | 9.6 | (8.3, 10.9) | 0.0077 | 10.6 | (6.1, 15.2) | 8.0 | (6.3, 9.7) | 0.2492 |
| ^a^Those in the flavored condition were shown 12 packages of cigarillos that are marketed with an explicit or implicit flavor; those in the unflavored condition were shown 12 packages of cigarillos that are not marketed with an explicit or implicit flavor.  ^b^ Use within the past 30-day.  ^c^ Based on bivariate analyses using t-tests.  ^d^ The mean represents the average proportional dwell time measured as the sum of total dwell time spent within the specified area of interest divided by the total amount of time the image was displayed. | | | | | | | | | | |
